# Probing the stability landscape of cylindrical shells for buckling knockdown factors

**DOI:** 10.1098/rsta.2022.0032

**Published:** 2023-04-03

**Authors:** R. M. J. Groh, A. Pirrera

**Affiliations:** Bristol Composites Institute, Department of Aerospace Engineering, University of Bristol, Bristol BS8 1TR, UK

**Keywords:** buckling, localization, nonlinearity

## Abstract

The buckling response of axially compressed cylindrical shells is well known for its imperfection sensitivity. Mapping out a stability landscape by localized probing has recently been proposed as a rational means for establishing shell buckling knockdown factors. Probing using a point force directed radially inwards and perpendicular to the cylinder wall is based on the insight that a localized single dimple exists as an edge state in the basin boundary of the stable prebuckling equilibrium. Here, we extend the idea of probing to bi-directional inwards and outwards forces to trigger both single-dimple and double-dimple edge states. We identify key features of the ensuing probing stability landscape and generalize these to derive three design curves of varying conservatism that are a function of the non-dimensional Batdorf parameter only. Interestingly, the most conservative of the three knockdown curves bounds a large dataset of experimental buckling results from below, despite being derived from probing features of geometrically perfect cylinders. Overall, the three design curves permit a more nuanced design approach than legacy knockdown factors, as different levels of conservatism can be chosen based on expected manufacturing quality. For instance, the most and least conservative of the three design guidelines differ by a factor of 3 for the most slender cylinder geometries, and the associated reduction in safety factor has profound implications for efficient structural design.

This article is part of the theme issue ‘Probing and dynamics of shock sensitive shells’.

## 1. Introduction

The buckling response of thin-walled shells is well known for its stochastic nature. Here, we focus on the axially compressed cylindrical shell, a common lightweight structural component in diverse engineering applications ranging from grain silos and water towers to space launch vehicles and aircraft fuselages. Copious tests on axially compressed thin-walled cylinders have demonstrated a large scatter in experimental buckling loads ranging from 10% to 90% [1] of the classical prediction derived from small-deflection theory [2]:

$$P_{\mathrm{cl}}=\frac{2\pi Et^{2}}{\sqrt{3(1-\nu^{2})}}\;\mathrm{and}\;u_{\mathrm{cl}}=\frac{Lt}{R\sqrt{3(1-\nu^{2})}},$$

where $P_{\mathrm{cl}}$ and $u_{\mathrm{cl}}$ are, respectively, the critical compression force and end-shortening of an isotropic (Young’s modulus E and Poisson’s ratio ν) cylinder of radius R, length L and wall thickness t. For imperfect cylinders the classical prediction overestimates the buckling load, creating unique challenges for safe design.

The discrepancy between classical prediction and experiments has been explained by various deficiencies in the analytical approach such as unrealistic boundary conditions, the role of nonlinearity in the prebuckling response, inevitable geometric imperfections during manufacturing and loading imperfections during testing [3–6]. Hence, accurately predicting the buckling load of compressed cylinders is challenging as the buckling load depends acutely on the precise nature of the initial, and potentially evolving, system conditions [7].

Today, these deficiencies can be accounted for by using nonlinear methods, computational models, and stochastic approaches, often leading to excellent correlation between predictions and experiments when all imperfections are accurately modelled. Unfortunately, in the design of new structures, the precise nature of manufacturing imperfections and in-service perturbations is not known. Therefore, the question remains of how best to design thin-walled shell structures without knowing the type, shape, and magnitude of imperfections and perturbations encountered in service.

Historically, this question has been addressed empirically relying on lower-bound curves to datasets of experimental results, e.g. NASA’s SP-8007 design guideline [8]. These design curves are also known as knockdown factors (KDFs) as they ‘knock down’ the classical prediction to a lower, more conservative value. With modern high-precision manufacturing processes, these historical design curves have become exceedingly conservative, limiting our ability to innovate and design more efficient structures [9]. In addition, legacy design curves do not permit a nuanced approach whereby a structural engineer chooses from different design curves of varying conservatism depending on expected levels of imperfection, manufacturing quality, operating environment, loading conditions, etc.

To this end, the idea of locally ‘probing’ a shell with an external poker to derive new and less conservative design guidelines has gained traction [10,11],^1^ albeit in various different manifestations. Central to all approaches is the realization—obtained through experimental observation and numerical modelling—that a localized dimple is a ‘stimulating, realistic and worst-case imperfection’ [13]. Although the critical instability point on the prebuckling path features a spatially periodic buckling eigenmode, the unstable postbuckling response of shells is generally governed by immediate localization [14]. High-speed photography experiments dating back to the 1970s confirm this picture [12,15], whereby the dynamic buckling event shows the formation of a single buckle that then propagates circumferentially (and often axially) over a time frame of milliseconds to restabilize in a diamond-shaped postbuckling mode. Through detailed computational studies [16–19], this dynamic pattern formation is now known to be an example of homoclinic snaking (also known as cellular buckling) [20] that also governs the evolution of complex patterns in other domains ranging from nonlinear optics and chemistry to convection [21].

In a snaking system, the localized building block of pattern formation forms the lowest saddle point in the energy landscape between a homogeneous state before pattern formation and a periodic state after pattern formation. Hence, the unstable single dimple is the saddle point of smallest energy—the mountain pass state—adjacent to the stable prebuckling equilibrium, and separates the unbuckled state from another stable postbuckled equilibrium [22]. From the perspective of nonlinear dynamics theory, the single dimple is the lowest-energy state on the basin boundary surrounding the prebuckling energy well [23]. Even for a theoretically perfect cylinder, the energy barrier that needs to be overcome to push the cylinder onto this single-dimple edge state is a small fraction of the strain energy stored in the unbuckled cylinder [17]. Furthermore, the more slender the cylinder (increasing L/t or R/t), the smaller the ratio between mountain pass energy and energy stored in the unbuckled shell [22], explaining the increased imperfection sensitivity of more slender shells. In summary, once the mountain pass state exists in the energy landscape surrounding the stable prebuckling equilibrium, the cylinder is balanced in a precarious ‘shock-sensitive’ state [10], where the cylinder can readily be triggered to buckle by external perturbations, or alternatively, be expected to buckle prematurely if initial imperfections erode the energy barrier even further.

Given the importance of the single dimple, a number of numerical and experimental procedures have been devised to stimulate its formation in an attempt to: (i) derive new knockdown factors for design, or (ii) create new testing methodologies. One class of approaches originates with the work of Hühne *et al.* [24,25], whereby a radial perturbation load of nominal magnitude is first applied to the cylinder and axial compression then increased until buckling occurs. Depending on where the perturbation is applied along the cylinder length and on the nature of the load (force or displacement controlled), different testing modalities can be applied [26]. In general, however, the buckling load first decreases with increasing perturbation magnitude before reaching a plateau. By interpreting this plateau as a worst-case knockdown in buckling load, a new design guideline relating KDF to cylinder geometry Batdorf parameter, $Z=(L^{2}/Rt)\sqrt{\left(1-\nu^{2}\right)}$ has been derived [27–29]. These design curves are less conservative than NASA’s SP-8007 guideline and knockdown factors derived from eliminating the membrane stiffness of a cylinder entirely [30]. The concept of perturbing a cylinder with point forces has also been extended to multiple, simultaneously applied perturbation loads [31], which produces greater knockdown in buckling load.

**Figure 1.**
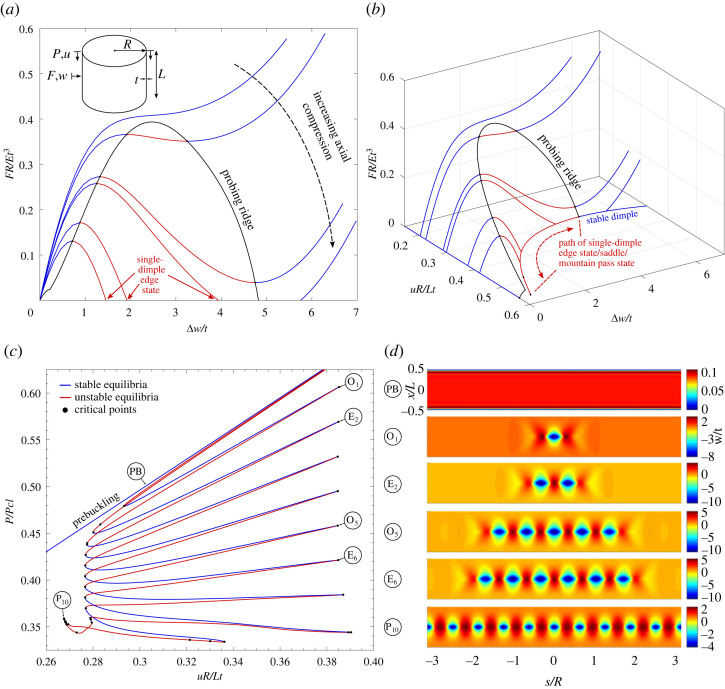
(*a*) Radially inwards probing force, F, versus incremental radial probing displacement, Δw, for different levels of axial end-shortening, u, in the prebuckling regime. Note, each prebuckling state has radial Poisson dilation, $w_{0}$, before poking commences. (*b*) 3D view of the probing stability landscape with the additional axis of axial end-shortening, u. Key features such as the ridge of turning points in the probing force–displacement paths, the unstable single-dimple solution (mountain pass state), and the stable single-dimple solution for F=0 are annotated. (*c*) Snaking equilibrium paths of axial reaction force, P, versus end-shortening, u, describing circumferential pattern formation. Note, the unstable segments of the single-dimple and double-dimple solutions run almost coincident to the prebuckling solution. (*d*) Radial deformation modes showing the sequence of multiplying odd- and even-numbered buckles that start from the single dimple and the double dimple, respectively, and both terminate in a periodic 10-buckle pattern. (Online version in colour.)

A more recent perturbation approach based on the concept of ‘shock sensitivity’ does not apply the radial perturbation load *a priori*, but probes and unprobes the cylinder repeatedly at different levels of axial compression [11]. The purpose of the probing procedure is manifold. By repeating the probing procedure for different levels of axial compression, a stability landscape is mapped out that shows a number of important features [32] (figure 1*a*,*b*). For low levels of axial compression, the probing force versus probing displacement curves are sigmoidal of exclusively positive stiffness. For intermediate values of compression, the curves develop maximum and minimum turning points in the sigmoidal probing force versus probing displacement curves, but crucially, the probe force never intersects the zero force axis for non-zero probing displacement. Above a critical value of axial compression, the probing force dips below the zero force axis, meaning that the induced single dimple now exists as an unstable equilibrium in addition to the stable prebuckling state. The work done by the probing poker in pushing the cylinder out of the stable prebuckling energy well and onto the saddle of the single dimple is a measurement of the energy barrier to buckling [10].

Another application of the probing procedure is in non-destructively predicting the buckling load of a manufactured cylinder [23]. The maximum turning points in the probing force versus probing displacement curves measured at various levels of axial compression form the so-called ‘ridge’ (figure 1*a*,*b*). By extrapolating the ridge to the level of axial compression where the probing force equals to zero, the buckling load of a tested cylinder can be estimated without inducing buckling. The challenge inherent in this approach is that the stiffness to probing—and therefore the maximum turning point in the probing force versus probing displacement curves—will, by definition, only fall to zero if the deformation mode induced by probing does indeed correspond to the natural buckling mode of the cylinder. Hence, if probing is not conducted where the single dimple would naturally develop under axial compression (a function of the imperfection signature; specifically, the location of the *sharpest* imperfection [7,33]), then ridge tracking will overestimate the buckling load [34].

A final application of the probing procedure is to use specific features of the stability landscape to derive non-empirical shell buckling knockdown factors. For example, in previous work the present authors have tracked the limiting level of axial compression for which the single dimple first exists as an unstable edge state with respect to varying geometric parameters (R, L, t) of the cylinder [17]. In doing so, a power-law relationship between cylinder geometry (Batdorf parameter, Z) and the onset of ‘shock sensitivity’ was derived computationally that bears striking similarities with the KDF proposed by Wagner *et al.* [27–29] using perturbation approaches, and that of Evkin *et al.* [35,36] using Pogorelov’s geometrical method (see [37] for a comparison). Hence, despite the different means of arriving at a single-dimple-based KDF, it is encouraging to note the close correlation of the different design curves.

The purpose and novelty of the present contribution is to extend the authors’ previous work on tracking key features of the stability landscape with respect to cylinder geometry [17]. In particular, we demonstrate that the cusp of the probing ridge, i.e. the point for which the probing force versus probing displacement curves are sigmoidal yet of exclusively positive stiffness (no turning point), represents the smallest level of compression for which a cylinder can buckle for any dimple imperfection magnitude. In addition, we extend the idea of probing to also poking the cylinder radially outwards. Probing outwards produces an outwards crest in the cylinder wall separating two adjacent inwards dimples. In previous work, we have demonstrated the importance of the double dimple in representing the dual solution to the single dimple in the homoclinic snaking sequence [18]. Indeed, the single-dimple and double-dimple solutions are the fundamental building blocks for pattern formation in the axially compressed cylinder and produce intertwined equilibrium paths of multiplying odd- and even-numbered buckles around the cylinder circumference (figure 1*c*,*d*). Hence, by tracking both the limit points of the single- and double-dimple solutions, and the cusps of the inwards and outwards probing ridges, we derive different cylinder buckling design curves of varying conservatism. Ultimately, this facilitates a more nuanced design approach where structural engineers have the option of choosing the level of conservatism depending on their expected level of cylinder perfection. For cylinders of large Batdorf parameter, which have the greatest sensitivity to imperfections, the difference in KDF between the least and most conservative design curves is a factor of 3. Hence, the choice of design curve can have profound implications for lightweight design of future engineering structures.

The rest of the paper is structured as follows. Section 2 introduces some of the key concepts of the probing stability landscape using the two-dimensional system of a compressed beam on a nonlinear elastic foundation, and particularly highlights the importance of the cusp of the probing ridge in determining the worst-case imperfect buckling response. Section 3 then extends the analysis to buckling of an axially compressed cylinder and presents several knockdown curves of varying conservatism derived from key features of the stability landscape. Finally, conclusions are drawn in §4.

## 2. Beam on a nonlinear elastic foundation

To introduce key concepts that are subsequently used to derive knockdown factors for axially compressed cylindrical shells, we first consider the stability landscape of a simpler two-dimensional system. A beam resting on and attached to a nonlinear elastic foundation has previously been used to explore the mechanics of shell buckling. Indeed, the rationale for using the beam on an elastic foundation to explore cylindrical shell buckling was initially formulated by von Kármán *et al.* [38]. If we imagine a cylindrical shell as a collection of axial strips, then the azimuthal curvature of the cylinder acts as a nonlinear foundation. Specifically, the linear effect of azimuthal curvature is to stiffen each axial strip transversely, i.e. to increase the buckling load, thereby reflecting membrane-bending coupling in the shell, while the quadratic effect reflects an asymmetry in transverse stiffness for larger deflections, i.e. radially inwards perturbations induce less strain energy than radially outwards perturbations. Thus, von Kármán *et al.* [38] showed experimentally that a beam resting on a nonlinear elastic foundation, where the foundation stiffness decreases for finite deflections, exhibits subcritical buckling with imperfection sensitivity.

In addition, the similarities between cylindrical shell buckling and the beam on a nonlinear elastic foundation go beyond what was initially anticipated by von Kármán *et al*. If the elastic foundation features initially destabilizing and finally restabilizing nonlinear terms, the beam first buckles subcritically and then restabilizes for finite deformations. Indeed, if the beam is sufficiently long, then it displays a tendency to first localize in one or multiple buckles which then multiply in a cellular manner along the length of the beam [20]. Thus, a foundation with quadratic and cubic nonlinear terms in the force–elongation response is sufficient to display a rich behaviour of pattern formation reminiscent of the axially compressed cylinder.

Here, we model an initially flat beam of bending rigidity EI and length L, resting on an elastic foundation characterized by a force, F, versus displacement, v, relationship $F(v)=k_{1}v+k_{2}v^{2}+k_{3}v^{3}$. The beam is simply supported at either end and loaded axially by a force P (compressive force taken as positive) to produce an end-shortening u. The critical buckling load is a function of the bending rigidity and the linear stiffness term of the foundation, giving the classical expression $P_{\mathrm{cr}}=2\sqrt{k_{1}EI}$ with a periodic, sinusoidal eigenmode of wavelength $l_{w}=2\pi {\left(EI/k_{1}\right)}^{0.25}$. The relative magnitude of the nonlinear terms $k_{2}$ and $k_{3}$ then governs the interplay between initial destabilization and later restabilization, and in particular, if cellular buckling (homoclinic snaking) occurs. Indeed, if $k_{2}\gg k_{3}$, then the destabilizing term dominates, and while the buckling mode will localize, the buckled beam does not restabilize for positive (compressive) values of P. Equally, if $k_{3}\gg k_{2}$, then the beam restabilizes almost instantly after buckling in a benign subcritical manner, precluding any opportunity for cellular buckling (snaking). Thus, both terms $k_{2}$ and $k_{3}$ (chosen in appropriate proportion) are required to observe imperfection-sensitive, localized buckling with pattern formation through snaking as observed in the cylindrical shell.

For algebraic simplicity, we assume $k_{1}=1$ and EI=1. To allow for cellular buckling, a sufficiently long beam of L=25π is assumed with $k_{2}=-55$ and $k_{3}=1000$. Using a finite-element formulation, the beam is discretized into 100 three-noded (quadratic) beam elements based on Reissner’s planar, large-displacement, finite-strain beam theory [39]. In deriving the internal force vector and tangent stiffness matrix of the beam element, the total potential energy is modified to include the potential of the foundation. As such, the foundation is not modelled explicitly as separate nonlinear spring elements attached to the beam, but rather the foundation restraint is subsumed into the element formulation of the beam directly. The resulting nonlinear system of equations is solved using an in-house generalized path-following solver that extends the capabilities of typical Riks path-following to include pinpointing of singular points (bifurcation points and limit points), branch switching at bifurcations, and tracking of singular points with respect to simultaneously varying parameters; see [40] for details.

### (a) Cellular buckling and mountain pass state of the perfect beam

**Figure 2.**
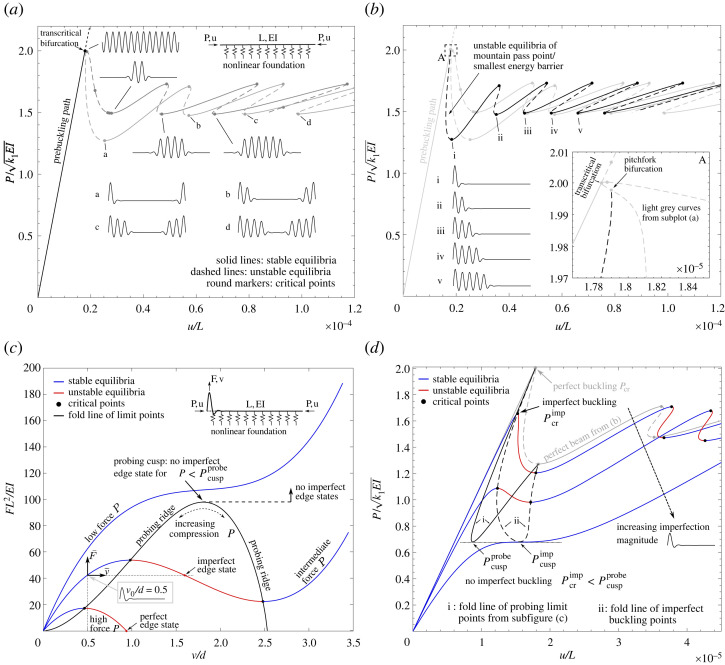
Mechanical response of an axially compressed beam resting on a nonlinear (softening/restiffening) elastic foundation. (*a*) The prebuckling path of axial force, P, versus end-shortening, u, destabilizes at a transcritical bifurcation at the classical load, $P_{\mathrm{cr}}=P/\sqrt{k_{1}EI}=2$. The two branches of the transcritical bifurcation are both snaking equilibrium paths that represent the multiplication of two different localized modes: one mode localized at the beam mid-point and another localized at both beam ends. (*b*) A pitchfork bifurcation off the end-localized transcritical branch leads to a fully localized mode at either end of the beam that also multiplies through snaking. The initially unstable branch of this fully end-localized mode corresponds to the path of lowest-energy edge states, i.e. mountain pass states adjacent to the prebuckling equilibrium. (*c*) Informed by the mountain pass state, probing close to one end of the beam for different values of compression, P, results in the familiar probe force, F, versus probe displacement, v, stability landscape similar to figure 1*a*. When initial geometric imperfections affine to the mountain pass state are present, the origin of the probing equilibrium path shifts to the new axes $\bar{F}\text{--}\bar{v}$, leading to imperfect edge states ($\bar{F}=0$) that did not exist in the perfect case (F=0). (*d*) Equilibrium paths, P versus u, of an imperfect axially compressed beam for increasing imperfection magnitude of a fully end-localized mode. No imperfect buckling limit point, $P_{\mathrm{cr}}^{\mathrm{imp}}$, exists below the cusp of the probing ridge, $P_{\mathrm{cusp}}^{\mathrm{probe}}$. (Online version in colour.)

The axial force, P, versus end-shortening, u, equilibrium manifold characteristic of the perfect beam is shown in figure 2*a*. The initially flat beam buckles at the classical value of $P/\sqrt{k_{1}EI}=2$ with a sinusoidal eigenvector of $l_{w}=2\pi$ (12.5 full waves). The bifurcation is transcritical (one stable and one unstable branch bifurcate off the flat solution) due to the up-down displacement asymmetry inherent in the quadratic term of the foundation. The initially stable branch of the transcritical bifurcation soon destabilizes by passing a limit point and sees the mode shape localize at the beam’s mid-span. With increasing end-shortening, the applied load oscillates between two values (the pinning region [41]) with each ‘snake’ of the de- and re-stabilizing equilibrium path adding additional buckles to the left and right of the growing wavefront (cellular buckling). The second unstable branch from the transcritical bifurcation follows a similar snaking sequence, but the buckling mode first localizes at both beam ends before multiplying towards the beam mid-point (see modes a–d in figure 2*a*).

As shown in figure 2*b*, this latter branch of the transcritical bifurcation features another bifurcation point in the vicinity of the prebuckling path (see inset A in figure 2*b*). Branching at this pitchfork bifurcation onto a new unstable path causes the buckling mode to localize even further—from a localization at *both* ends to a single localization at *either* the left or the right end (see mode i in figure 2*b*). Crucially, this fully end-localized mode corresponds to the mountain pass state on the basin boundary surrounding the prebuckling energy well. Hence, even though there are four unstable branches shown in figure 2*b* running adjacent to the stable prebuckling path, the two overlapping branches (black dashed curve) representing a fully end-localized mode at either end of the beam correspond to the unstable equilibria of least energy for all levels of axial load above limit point i.

This observation can be confirmed by inspection, as the fully end-localized mode only features half the number of waves as the modes depicted in figure 2*a*, thereby storing less strain energy. We also confirmed this observation computationally by employing the perturbation energy (Störenergie) algorithm [42], which directly computes the smallest-energy edge state surrounding a stable equilibrium by means of a quadratic eigenvalue problem. The eigenvalue problem failed to converge for axial loads less than the limit point i, confirming that no edge states exist below the limiting axial force of the fully end-localized mode. Finally, figure 2*b* shows that for increasing end-shortening beyond limit point i, the equilibrium path undergoes the now familiar snaking sequence, leading to the propagation of the single localized buckle from one beam end to the other (see modes i–v shown in figure 2*b*).

Thus, a beam on a nonlinear elastic foundation of appropriately chosen elastic stiffness values features some of the key characteristics present in the axially compressed cylinder, including: (i) subcritical buckling; (ii) localization of buckling modes; (iii) cellular buckling/homoclinic snaking and (iv) a clearly defined mountain pass state.

### (b) Probing the perfect beam and imperfect beam buckling response

Based on this knowledge of the mountain pass state, we perform numerical probing experiments that trigger the associated localized mode. Hence, a point force is applied vertically to the beam at the node that lies closest to the peak of the fully end-localized mode (figure 2*c*). The probing procedure is conducted from different prebuckling states of varying axial load. Equilibrium curves of probing force, F, versus probing displacement, v,^2^ for three levels of axial load, P, are shown in figure 2*c*. The probing curves show the same qualitative behaviour as for the axially compressed cylinder in figure 1*a*. For low axial load, P, the probing curve is sigmoidal of exclusively positive stiffness. For intermediate levels of P, maximum and minimum turning/limit points appear on the probing curves, but the curves do not intersect the probing force axis, F=0. For high levels of axial load, i.e. when the end-localized mountain pass state is a self-equilibrated, yet unstable, equilibrium (P greater than the limit point i marked in figure 2*b*), the probing curves intersect the horizontal axis.

We now pose the question of what is expected to occur if initial geometric imperfections in the shape of the mountain pass state are present? We suggest that introducing an initial imperfection is, to first order, equivalent to shifting the origin of the probing curves, as shown by the $\bar{F}$–$\bar{v}$ axes in figure 2*c*. This shift in coordinate axes reflects the fact that both the magnitude of the probing force and the probing displacement required to obtain a specific deformation amplitude diminish with the presence of an affine imperfection. Depending on the magnitude of the initial imperfection, $v_{0}$, this implies that probing curves for the perfect beam ($v_{0}=0$) that did not intersect the F=0 axis may intersect the imperfect $\bar{F}=0$ axis, as shown in figure 2*c*. Hence, we obtain ‘imperfect’ edge states that did not exist for the perfect beam which, crucially, exist for lower levels of axial load, P. However, if the axial load is sufficiently small, such that probing of the perfect beam does not lead to maximum and minimum turning/limit points on the probing curves, then no level of initial imperfection can shift the $\bar{F}=0$ axis to yield an imperfect edge state. This transition is defined by the point where the probing ridge, i.e. the locus of maximum/minimum points on the probing curves, reaches a turning point—a so-called codimension-2 cusp catastrophe. Thus, for levels of axial compression, P, below the axial force, $P_{\mathrm{cusp}}^{\mathrm{probe}}$, of the probing ridge cusp of the perfect beam, imperfect edge states are not possible.

This behaviour is confirmed in figure 2*d*, which shows the P–u equilibrium path of the perfect beam (in grey) in addition to various equilibrium paths of imperfect beams with increasing magnitude of the fully end-localized, mountain-pass-state imperfection. As expected, with increasing imperfection magnitude, the prebuckling equilibrium curves are rounded off and the bifurcation of the perfect system is converted into a limit point that induces buckling at a lower axial load, $P_{\mathrm{cr}}^{\mathrm{imp}}$, than the bifurcation load of the perfect system, $P_{\mathrm{cr}}$. The description of the previous paragraph implies that beams with fully end-localized initial imperfections should exhibit unstable edge states for levels of axial load, P, well below those of the perfect beam. Indeed, this is precisely what is observed in figure 2*d*. In addition, the locus of imperfect limit points (black dashed curve) in figure 2*d* shows that above a threshold value of imperfection magnitude, the limit points in the imperfect equilibrium curves vanish entirely at another cusp catastrophe. As a result, all imperfect beams buckle for axial loads greater than this cusp load, $P_{\mathrm{cusp}}^{\mathrm{imp}}$. At this stage, the behaviour of the beam is best described as having a new, non-flat geometry with the undulation of the end-localization acting as a governing feature, rather than a ‘quasi-perfect’ beam with an initial imperfection.

Interestingly, the value of $P_{\mathrm{cusp}}^{\mathrm{imp}}$ is slightly greater than the load, $P_{\mathrm{cusp}}^{\mathrm{probe}}$, at the cusp of the probing ridge of the perfect beam (solid black curve in figure 2*d*). Based on interrogating the probing landscape in figure 2*c* and the buckling response of imperfect beams in figure 2*d*, we therefore argue that a beam with an imperfection affine to the mountain pass state cannot buckle at loads lower than $P_{\mathrm{cusp}}^{\mathrm{probe}}$. Indeed, by conducting other single-point and multi-point force probing experiments on the perfect beam, we have found that probing to induce the mountain pass state leads to the lowest value of $P_{\mathrm{cusp}}^{\mathrm{probe}}$. In addition, by running a large set of localized, random, and periodic sinusoidal imperfection signatures, we could not determine an imperfect buckling load, $P_{\mathrm{cr}}^{\mathrm{imp}}$, smaller than $P_{\mathrm{cusp}}^{\mathrm{probe}}$, with fully end-localized imperfections causing the most severe knockdown.

We argued previously that introducing an initial imperfection is, to first order, equivalent to shifting the origin of the probing curves in the probing landscape of figure 2*c*. Hence, the probing displacement at the probing cusp of the perfect beam should provide a first-order approximation of the initial imperfection magnitude required to minimize the imperfect buckling load. The imposed imperfection magnitude (measured as the greatest lateral deflection $v_{0}$ from the initially flat beam) that minimizes the imperfect buckling load, $P_{\mathrm{cusp}}^{\mathrm{imp}}$, in figure 2*d* is $v_{0}/d=2.33$. Conversely, the lateral deflection at the probing cusp of the perfect beam, $P_{\mathrm{cusp}}^{\mathrm{probe}}$, shown in figure 2*c* is v/d=1.81. The nonlinearity of the problem precludes the possibility of a perfect mapping of the displacement at the probing cusp (based on the perfect beam) to the imperfection magnitude of the lowest buckling load (relating to an imperfect beam). However, our analyses suggest that the probing cusp displacement (v/d=1.81) can be used as a *conservative* estimate of the imperfection magnitude ($v_{0}/d=2.33$) required to minimize the buckling load with a mountain-pass-affine imperfection mode.

In summary, we have shown that a geometric imperfection affine to the mountain pass state leads to the largest knockdown in buckling load. Beyond a certain threshold magnitude of imperfection, the limit point instability that defines imperfect buckling vanishes and the beam buckles at a higher load. However, this comes at the cost of a significantly altered, lower-stiffness prebuckling response, and as a result, the beam is best interpreted as a new structure of altered geometry rather than a nominally flat beam with imperfections. Interestingly, the smallest possible buckling load of the *imperfect* beam can be determined by a probing procedure on the *perfect* beam that induces the fully end-localized mountain pass state. Specifically, the cusp on the probing ridge of the stability landscape is a good proxy for the lowest imperfect buckling load.

These properties of the probing stability landscape are equally valid for other systems such as the axially compressed cylinder. The beam on a nonlinear elastic foundation was analysed in this section due to its greater simplicity while maintaining close mechanical similarity to the cylindrical shell in subcritical buckling, localization, snaking and the probing landscape. This leads, in our opinion, to a much neater exposition of the key concepts summarized above. In the following sections, we use the identified properties of the probing stability landscape to determine a lower-bound buckling load for axially compressed, geometrically imperfect cylinders.

## 3. Axially compressed cylindrical shell

As a baseline model, we take the longest cylinder considered in Yamaki’s seminal experiments [43]. Hence, we consider a thin-walled shell of wall thickness t=0.247 mm, mid-thickness radius R=100 mm and axial length L=160.9 mm. As Yamaki’s cylinders were manufactured from mylar, we assume the constitutive behaviour is isotropic and linear elastic under the strain regime considered, with Young’s modulus E=5.56 GPa and Poisson’s ratio ν=0.3.

The cylinder is discretized into isoparametric, geometrically nonlinear finite elements based on a total Lagrangian formulation. The finite elements used are the so-called ‘degenerated shell elements’ [44] based on the assumption of the first-order shear deformation theory [45] (shear correction factor k=5/6). The full cylinder is discretized into 193 axial and 480 circumferential nodes resulting in 463 200 degrees of freedom (d.f.) that are assembled into 25-noded *hp*/spectral finite elements (48 axial and 120 circumferential elements) using the interpolation scheme by Payette & Reddy [46]. To mirror Yamaki’s experiments, all nodes at the top and bottom ends of the cylinder are clamped, with all d.f. constrained apart from the applied axial end-shortening displacement, u. The elements are fully integrated, and the effects of shear and membrane locking are minimized by the use of bi-quartic isoparametric interpolation functions (25-noded elements). The chosen mesh density and modelling approach was previously validated by excellent correlation with Yamaki’s experimental results [18]. The shell element is implemented in the same in-house FE code and nonlinear solver as used in §2 (see Groh *et al.* [40] for full capabilities).

### (a) Stability landscape of radially inwards and outwards probing

**Figure 3.**
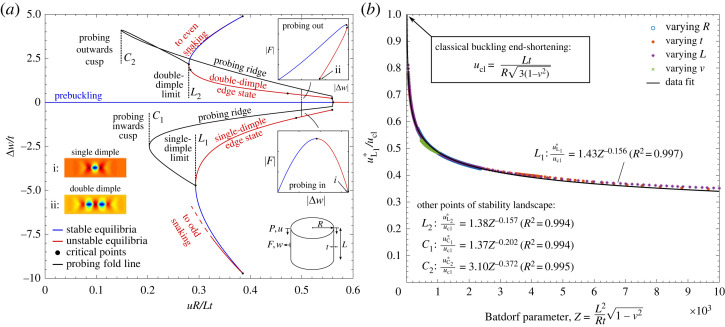
(*a*) Probing stability landscape in terms of axial end-shortening, u, versus radial probing displacement, Δw, for probing radially inwards and outwards. Probing inwards leads to the single-dimple edge state while probing outwards leads to the double-dimple edge state. The limiting level of axial end-shortening for which the single dimple and the double dimple exist as edge states are denoted as limit points $L_{1}$ and $L_{2}$, respectively. The level of axial end-shortening for which probing inwards and outwards results in a sigmoidal probing force versus probing displacement curve of exclusively positive stiffness (no turning points) are denoted by the cusps $C_{1}$ and $C_{2}$, respectively (cf. figure 2*c*). (*b*) The axial end-shortening, $u^{*}$, of the four points $L_{1}$, $L_{2}$, $C_{1}$ and $C_{2}$ can be generalized for any cylinder by tracking these points with respect to varying model parameters R, t, L and ν. The data points of $u_{L_{1}}^{*}$ for point $L_{1}$ versus the non-dimensional Batdorf parameter, Z, collapse onto one curve. A least-squares power law shows excellent fit to these data. The fitted power-law curves for the other points $L_{2}$, $C_{1}$ and $C_{2}$, obtained using the same methodology, are also stated. (Online version in colour.)

Figure 3*a* shows the probing stability landscape in terms of controlled end-shortening, u, versus the incremental radial deflection, Δw, measured at the cylinder mid-length (probing location is invariant to azimuthal coordinate). Note that on the prebuckling path the cylinder dilates as a result of Poisson’s expansion. Towards the two ends of the cylinder this leads to the well known boundary layer (see mode PB in figure 1*d*), but towards the cylinder mid-length the radial expansion is uniform and a function of the applied end-shortening, i.e. $w_{0}=w_{0}(u)$. The incremental variable Δw(u) therefore expresses the deviation from the prebuckling radial deflection as a function of u.

In figure 3*a*, the prebuckling path is shown as the horizontal curve Δw(u)=0. The prebuckling solution loses stability at a bifurcation point for uR/Lt=0.561, which is 92.7% of the classical buckling end-shortening, $u_{\mathrm{cl}}=Lt/\left(R\sqrt{3(1-\nu^{2})}\right)$. This 8% knockdown to the classical end-shortening occurs as a result of the nonlinear boundary layer towards the clamped edges, which is not accounted for in the classical model. Also shown in figure 3*a* are the equilibrium paths of the single-dimple and double-dimple solutions determined in previous work [18], which correspond to a locus of zero probing force, F=0. The displacement norm Δw describes the incremental radial displacement at the centre of the single buckle of the single-dimple solution and at the centre of the crest separating the two buckles of the double-dimple solution. As the single-dimple displacement is radially inwards (see mode i in figure 3*a*), the abscissa is negative. For the double dimple, the displacement Δw is positive because an outwards crest separates the two adjacent inward buckles (see mode ii in figure 3*a*).

Both single-dimple and double-dimple solutions are initially unstable and therefore represent subcritical buckling behaviour. The single-dimple and double-dimple solutions then stabilize at limit points $L_{1}$ and $L_{2}$, respectively, where they go onto the odd and even snaking sequences, respectively, depicted in figure 1*c*. A single-dimple edge state for a specific level of end-shortening can be found by probing into the shell (Δw<0) using a point force centred at the cylinder mid-length. A projection of an inwards probing path is shown for uR/Lt=0.5 in the u–Δw plane, and the familiar probing path in terms of probing displacement, Δw, versus probing force, F, is shown as an inset. The single-dimple edge state is obtained when F=0.

Also shown in figure 3*a* is a radially outwards probing path for the same end-shortening uR/Lt=0.5. In previous experimental and numerical work, only radially inwards probing was considered [17,23,32,34], but its dual—namely, radially outwards probing—is equally informative as it leads to the double-dimple edge state. The top-right inset of probing displacement, Δw, versus probing force, F, shows a similar inverted U-shaped curve with a maximum turning/limit point. Probing radially outwards forces an outwards crest into the cylinder wall with two dimples appearing on either side of the crest. The double-dimple edge state is obtained when F=0.

Hence, both probing inwards and outwards is important as both the single dimple and the double-dimple represent mountain pass states for different regimes of end-shortening, u, and either probing setup can be used to effectively trigger the corresponding deformation modes. As established by Horák *et al.* [22], the single dimple is a mountain pass state adjacent to the prebuckling energy well, but as the single-dimple solution vanishes for end-shortening less than limit point $L_{1}$, i.e. $u<u_{L_{1}}^{*}$, the double-dimple solution then takes over as the mountain pass state between limit points $L_{1}$ and $L_{2}$, i.e. $u_{L_{2}}^{*}<u<u_{L_{1}}^{*}$. Thus, for a complete picture of the probing stability landscape both inwards and outwards probing is necessary, an insight that is currently lacking in the shell-probing literature. Also note that henceforth the end-shortening values corresponding to critical points of interest are denoted by a star superscript.

Apart from determining $L_{1}$ and $L_{2}$, the other features of the probing stability landscape worth noting are the cusps of the probing ridges $C_{1}$ and $C_{2}$. The two solid black curves in figure 3*a* denote the locus of the maximum and minimum turning points on the probing Δw–F curves with changing u, i.e. the probing ridges for radially inwards and outwards probing. As discussed in the previous section, the cusp of the probing ridge denotes the lowest buckling end-shortening for a cylinder with an imperfection affine to the probing deformation mode. Hence, the end-shortening at $C_{1}$ (uR/Lt=0.203) provides the minimum buckling load of a cylinder with a single-dimple imperfection (mode i in figure 3*a*), whereas $C_{2}$ (uR/Lt=0.148) provides the minimum buckling load of a cylinder with a double-dimple imperfection (mode ii in figure 3*a*).

We have therefore identified four key features of the probing stability landscape:

— limit points $L_{1}$ and $L_{2}$, which denote, respectively, the smallest level of compression for which the single-dimple and double-dimple edge states exist.
— cusp points $C_{1}$ and $C_{2}$, which denote, respectively, the smallest level of compression for which a cylinder with single-dimple or double-dimple imperfection can buckle.

The significance of limit points $L_{1}$ and $L_{2}$ is that once the single dimple or the double dimple exist as edge states in the equilibrium manifold, the cylinder is in a heightened state of ‘shock sensitivity’ [10]. For $u>u_{L_{2}}^{*}$, even small external perturbations can trigger an escape out of the prebuckling energy well, over the mountain pass point, and into the postbuckling regime. Hence, limit points $L_{1}$ and $L_{2}$ denote safe, lower-bound compression loads for ‘quasi-perfect’ cylinders that have been manufactured with great care to tightly dimensioned tolerances. Cusp points $C_{1}$ and $C_{2}$ generalize this notion to imperfect cylinders; the cusps provide a lower-bound threshold below which a cylinder with certain finite imperfections can no longer lose stability.

In previous work by Gerasimidis *et al.* [47], the inwards probing cusp of the stability landscape was interpreted slightly differently as denoting the minimum compression level for which a force-controlled perturbation radially into a *perfect* cylinder could lead to buckling. This interpretation is certainly correct, but our results (figure 2*c*) show that the significance of the cusp point goes beyond this interpretation. Namely, the cusp in the probing landscape of the *perfect* cylinder is of significance for the behaviour of *imperfect* cylinders as well. This is because limit point $L_{1}$ of an imperfect cylinder (denoting the onset of the single-dimple edge state) tends to $C_{1}$ of the perfect cylinder as a single-dimple imperfection magnitude is increased. The same is true for $L_{2}$ and $C_{2}$ in terms of the double-dimple edge state and increasing magnitude of a double-dimple imperfection. Our insight into cusp point $C_{1}$ and its dual $C_{2}$ thus generalizes their interpretation, and thereby the notion of shock sensitivity, from the perfect into the imperfect regime. Overall, compared to the current consensus in the probing literature, consideration of the cusp points and bi-directional inwards and outwards probing significantly extends our perspective of using the stability landscape to predict buckling.

### (b) Cylinder buckling knockdown factors from the stability landscape

Of course, the stability landscape in figure 3*a* is only valid for one cylinder (t=0.247 mm, R=100 mm, L=160.9 mm, $Z=L^{2}\sqrt{1-\nu^{2}}/Rt=1000$). To generalize the response to other cylinder geometries we use the critical-point-tracking capability of our in-house generalized path-following code to track $L_{1}$, $L_{2}$, $C_{1}$ and $C_{2}$ through parameter space. In particular, we follow the evolution of these points with respect to thickness, t, radius, R, axial length, L and Poisson’s ratio, ν. A dataset of limit point $L_{1}$ for each of the four varying parameters is shown in figure 3*b* in terms of the normalized end-shortening at the limit point, $u_{L_{1}}^{*}/u_{\mathrm{cl}}$, versus the Batdorf parameter, Z. Note that the ratio $u_{L_{1}}^{*}/u_{\mathrm{cl}}$ can be interpreted as a knockdown factor as it expresses the onset of the single dimple as a mountain pass state as a ratio of the classical buckling end-shortening, $u_{\mathrm{cl}}$. Furthermore, for the case of elastic buckling and the quasi-linear prebuckling path considered here, the ratio $u/u_{\mathrm{cl}}$ along the prebuckling path is equivalent to $P/P_{\mathrm{cl}}$.

Interestingly, the computed set of more than 800 data points in figure 3*b* collapses onto a single curve that is accurately approximated by a power law $u_{L_{1}}^{*}/u_{\mathrm{cl}}=1.43Z^{-0.156}$ ($R^{2}=0.997$) fitted by a least-squares algorithm. Hence, as suggested in previous publications, e.g. [17,36,37], a knockdown curve derived from the single dimple is a function of the well-known Batdorf parameter that characterizes shell buckling. More importantly, the other three key features of the stability landscape $L_{2}$, $C_{1}$, and $C_{2}$ are also functions of the Batdorf parameter. Indeed, when tracking these three points with respect to t, R, L and ν, the computed sets of (multiple hundred) data points of the respective critical points, $u^{*}/u_{\mathrm{cl}}$, also collapse onto a single curve with respect to Z. The associated power laws computed by a least-squares algorithm are indicated in figure 3*b*.

We have reason to believe that these fitted power laws reflect more of the governing physics than a simple regression analysis might suggest. If the data points for varying thickness are isolated, then a power law of essentially perfect correlation can be fitted in all cases. For varying radius or length, most of the error in the conducted regressions arises from the extremes of the computed R and L values. This occurs because the changing planar geometry leads to a changing aspect ratio of the discretized finite elements with associated variations in numerical accuracy. We thus conjecture that the small root-mean-square error of the fitted power laws is by no means a statistical accident but reveals some of the underlying physics of the problem; at the very least, the importance of the Batdorf parameter in characterizing the probing stability landscape and imperfect buckling loads.

**Figure 4.**
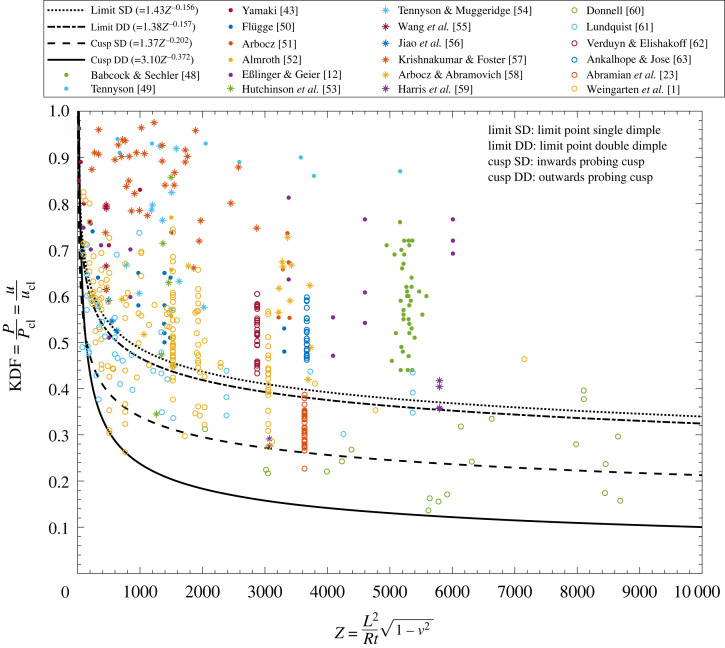
Dataset of 514 experimental buckling results from [1,12,23,43,48–63] plotted in terms of recorded KDF versus Batdorf parameter, Z. For comparison, the four design curves derived from the probing stability landscape are also shown, with the most conservative curve forming a lower-bound envelope to the experimental results. Note that NASA’s SP-8007 guideline approaches the same lower-bound KDF of 0.1 for large Z. (Online version in colour.)

In essence, each of the four power laws describes a different design guideline for cylinder buckling derived from the probing stability landscape. The curves are appropriate measures of knockdown factors (KDFs) as $u_{L_{1}}^{*}/u_{\mathrm{cl}}$ and $u_{L_{2}}^{*}/u_{\mathrm{cl}}$ describe the proportion of the classical load required to place even a geometrically perfect cylinder in a heightened shock-sensitive state. Equally, $u_{C_{1}}^{*}/u_{\mathrm{cl}}$ and $u_{C_{2}}^{*}/u_{\mathrm{cl}}$ capture the greatest knockdown in buckling load expected from single dimple and double dimple, i.e. mountain-pass-affine, imperfections. To test the four design guidelines against experimental data, each curve is plotted on one set of axes of Batdorf parameter, Z, against KDF ($u^{*}/u_{\mathrm{cl}}$) in figure 4.^3^ Also included in this figure are 514 data points taken from 20 studies spanning more than 100 years of cylinder buckling experiments [1,12,23,43,48–63]. As such, the data shown include modern experiments on tightly dimensioned cylinders [57], as well as earlier, less accurate experiments on cylinders manufactured by rolling sheet metal around a mandrel with a single axial weld line [60].

In general, the design curves can be split into three categories.

(i) The design curves derived from $L_{1}$ and $L_{2}$ are nearly identical, with the curve $u_{L_{2}}^{*}/u_{\mathrm{cl}}=1.38Z^{-0.157}$ denoting the more conservative of the two. This design guideline represents the threshold of end-shortening for which even a ‘quasi-perfect’ cylinder is in a precarious shock-sensitive state such that buckling is easily induced by external perturbations. This curve bounds 73.0% of the dataset shown in figure 4 from below and is the least conservative guideline presented here.
(ii) The design curve $u_{C_{1}}^{*}/u_{\mathrm{cl}}=1.37Z^{-0.202}$ corresponds to the lower-bound buckling load for a cylinder with a dominant single-dimple imperfection in the shape of the mountain pass state.^4^ This curve bounds 95.3% of the dataset shown in figure 4 from below and is of medium conservatism. It is recommended for cases where one dominant sharp imperfection or localized disturbing features are present to erode the buckling load. As identified for the beam on a nonlinear foundation, the probing displacement at the cusp point can be used as a conservative estimate of the initial imperfection magnitude required to reach the minimum threshold in imperfect buckling load. Defining the non-dimensional single-dimple amplitude as $(w/t)\sqrt{1-\nu^{2}}$, we have used the computed dataset of cusp point $C_{1}$ and the least-squares regression approach outlined above, to empirically determine a relationship between the dimple magnitude at $C_{1}$ and the Batdorf parameter:
  3.1 $$\frac{w}{t}\sqrt{1-\nu^{2}}=0.44Z^{0.24}\;(R^{2}=0.992).$$
  This relation thus provides a conservative estimate of the maximum allowable magnitude of a dimple-shaped imperfection for which the above design curve is applicable.
(iii) The design curve $u_{C_{2}}^{*}/u_{\mathrm{cl}}=3.10Z^{-0.372}$ provides the theoretical lower-bound buckling load of a cylinder under the current loading regime (clamped edges and displacement-controlled loading). This is because neither mountain-pass-affine imperfection of finite magnitude, shaped either as a single or a double dimple, can lead to buckling below the KDF suggested by this curve.^5^ Indeed, this curve bounds 99.2% of the dataset shown in figure 4 from below; in total, only four data points fall below the curve of which two points fall within 0.5% of the curve. This most conservative design guideline is therefore recommended when no prior information about initial imperfections exists. Interestingly, this design guideline approaches the same lower-bound KDF of 0.1 as NASA’s SP-8007 guideline [1]. NASA’s curve is not shown in figure 4 as it is a function of R/t rather than Z.

Hence, the three design curves are applicable under different scenarios and assumptions: near-perfect manufacturing quality and loading for curve (i); manufacturing within the tolerance of equation (3.1) for curve (ii); and a lower-bound threshold without assumptions on quality for curve (iii). For each category, the geometric parameter Z of a shell needs to be specified, and corrections to the classical buckling load can then be applied based on the desired level of conservatism. In this manner, the three different design curves (derived from $L_{2}$, $C_{1}$ and $C_{2}$) can be interpreted as varying levels of risk that an engineer can assume during design based on an assumption of manufacturing quality.

As is commonly the case, the flipside of risk is a commensurate level of reward, which in our case is the potential for lightweighting. For the most slender of designs (high Z), which are commonly encountered in the design of space launch vehicles, the most and least conservative of the three design guidelines can differ by a factor of 3 (KDF of 0.1 versus 0.3 for $Z=1\times 10^{4}$). The associated threefold reduction in safety factor can have profound implications on material usage, cost, and ultimately system performance, especially in industries such as space transportation where the payload cost to orbit is measured in $1000s per kilogram. Overall, the three design curves identified from $L_{2}$, $C_{1}$ and $C_{2}$ permit a more nuanced design approach than legacy knockdown factors, whereby structural engineers have the option of choosing from different design curves of varying conservatism depending on expected levels of imperfection, manufacturing quality, operating environment, loading conditions, etc.

## 4. Conclusion

Probing has emerged as a promising experimental and numerical evaluation technique for subcritical buckling. As such, it has been applied to cylindrical shells [32], cable-stayed columns [64], snap-through arches [65] and slender space structures [66]. The key requirement for a successful probing evaluation is that the applied perturbation induces the critical mode that governs the buckling instability. The method is particularly useful for systems prone to localized, rather than distributed, periodic buckling as the associated localized mode shape is readily controlled using localized actuation.

For cylindrical shells, the present and previous work [18] have identified the single-dimple and double-dimple localizations as governing instability modes. This is because beyond a critical threshold of end-shortening, both localizations represent edge states in the basin boundary surrounding the stable prebuckling equilibrium. Beyond this critical threshold, the cylindrical shell exists in a heightened state of ‘shock sensitivity’ [10], whereby small external perturbations can lead to premature buckling even for theoretically perfect shells. By mapping-out the stability landscape of these two localized modes using inwards and outwards probing forces, we have identified four key features of the stability landscape that form the basis for rationally derived knockdown factors (KDFs). These are: firstly, the end-shortening that denotes the onset of the single-dimple and double-dimple localizations as edge states; and secondly, the cusp points of the probing ridge that denote the lowest buckling load with either localized dimple mode as an initial imperfection of finite magnitude. Hence, interestingly, the lower-bound buckling load of an *imperfect* shell can be predicted from the probing response of the *perfect* shell, as long as probing is conducted to trigger the most critical of edge states (i.e. the mountain pass state). Indeed, this observation has placed new focus on exactly how a shell is probed; specifically, the importance of outwards probing to determine the double-dimple edge state. As of this writing, there is no experimental validation of outwards probing and its stability landscape, which may motivate new experiments to this effect.

We have identified three unique design curves derived from the probing stability landscape corresponding to varying levels of conservatism, with the most conservative forming a lower bound to 99.2% of a dataset comprising 514 experimental results taken from the literature that span more than a century of buckling experiments. As such, the knockdown factors presented here permit structural engineers to choose a preferred level of conservatism based on prior information of likely imperfection magnitudes: (i) a ‘quasi-perfect’ cylinder; (ii) a pronounced and dominating defect and (iii) the lower-bound response, i.e. no prior knowledge of imperfection type and magnitude exists. For the most imperfection-sensitive designs, i.e. those with large Batdorf parameter, the least conservative KDF allows for a threefold reduction in safety factor compared to legacy design guidelines (e.g. NASA’s SP-8007 guideline) with profound implications for more efficient and sustainable structural design.

All KDF derived herein assume the classical CC4 boundary condition (ends clamped and displacement-controlled compression) as this is the condition most commonly implemented in laboratory experiments. Future work will investigate the effect of changing boundary condition on the stability landscape, e.g. pinned ends and force-controlled compression.

In conclusion, we believe that the present work creates opportunities for more lightweight and higher-performing cylindrical shell structures in a broad range of engineering applications.

## Data Availability

Data are available at the University of Bristol data repository, data.bris, at https://doi.org/10.5523/bris.1ycd90k17l2tt25rs2upikl2nj [67].

## Footnotes

1 The idea of ‘probing’ can also be traced back to the seminal paper by Eßlinger & Geier [12], where cylinders were perturbed at different levels of axial compression by tapping with a finger.

2 The lateral displacement, v, is normalized by the diameter of the circular beam cross-section, d=0.01195.

3 Note that the Batdorf parameter can be rewritten as $Z={\left(L/R\right)}^{2}(R/t)\sqrt{1-\nu^{2}}$. The length-to-radius ratio of practical cylindrical shells encountered in industry is in the range L/R=0.5−5. Hence, assuming that L/R=O(1) for practical cylinders, Z has the same asymptotic behaviour as R/t. Large Z is thus equivalent to large R/t for practical cylinders.

4 A subtle difference between knockdown curves derived from cusp points $C_{1}$ (single dimple) and $C_{2}$ (double dimple) is that the former assumes that one localized (or multiple separated localized imperfections that do not interact) are present, whereas the latter is derived from the possibility that two or more adjacent imperfections interact.

5 Note that for very short cylinders, Z<100, the design curve derived from $C_{1}$ (the single-dimple cusp) is more conservative than the design curve derived from $C_{2}$ (the double-dimple cusp).
